# Disorder‐to‐order transition in PE–PPE proteins of *Mycobacterium tuberculosis* augments the pro‐pathogen immune response

**DOI:** 10.1002/2211-5463.12749

**Published:** 2019-12-17

**Authors:** Javeed Ahmad, Mohd Khubaib, Javaid Ahmad Sheikh, Rita Pancsa, Saroj Kumar, Alagiri Srinivasan, Mohan Madan Babu, Seyed E. Hasnain, Nasreen Z. Ehtesham

**Affiliations:** ^1^ Inflammation Biology and Cell Signalling Laboratory National Institute of Pathology New Delhi India; ^2^ Department of Biophysics All India Institute of Medical Sciences New Delhi India; ^3^ JH Institute of Molecular Medicine Jamia Hamdard New Delhi India; ^4^ Department of Biotechnology Jamia Hamdard New Delhi India; ^5^ Medical Research Council, Laboratory of Molecular Biology Cambridge UK; ^6^ Dr. Reddy's Institute of Life Sciences Hyderabad India; ^7^ Present address: Molecular Biology Section Laboratory of Immunology National Institute of Allergy and Infectious Diseases National Institutes of Health Bethesda MD 20892 USA

**Keywords:** folding‐upon‐binding, immune modulation, immunogenicity, *Mycobacterium tuberculosis*, pathogenesis, protein–protein interaction

## Abstract

A growing body of evidence supports the hypothesis that intrinsically disordered proteins often mediate host–pathogen interactions and modulate host functions for pathogen survival and virulence. *Mycobacterium tuberculosis* (*M.tb*) has evolved largely through reductive evolution, with a few exceptions such as the glycine–alanine‐rich PE–PPE/PGRS protein family, which has been expanding in pathogenic mycobacteria. Here, our analyses of the *M.tb* proteome and secretome revealed that the PE–PGRS subfamily is enriched for disordered regions and disordered binding sites, pointing to their importance in host–pathogen interactions. As a case study, the secondary structure of PE35–PPE68 and PE32–PPE65 of the pathogenesis‐related RD1 and RD8 regions was analyzed through Fourier‐transform infrared spectroscopy. These disordered proteins displayed a considerable structural shift from disordered to ordered while engaged in the formation of complexes. While these proteins are immunogenic individually and enhance the pro‐pathogen response, their corresponding complexes enhanced the responses manifold as displayed here by PE35 and PPE68. It is likely that *M.tb* exploits such disorder–order structural dynamics as a strategy to mount a pro‐pathogen response and subvert host defense for productive infection. This functional gain also serves as a means to compensate genomic content loss due to reductive evolution.

## Graphical abstract

*Mycobacterium tuberculosis *exploits disordered proteins to subvert the host immune response. Bioinformatic analysis of various protein families of *M. tuberculosis* revealed that PE/PPE–PGRS family proteins are highly disordered and interact with cognate partners to achieve ordered structure as validated by FTIR. Immunization of mice indicated that the ordered protein complex exhibits gain of function by augmenting the immune response compared to that produced against disordered proteins.

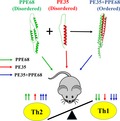

**Abbreviations**
BCG: bacillus Calmette–Guérin
BSA: bovine serum albumin
*E. coli*: *Escherichia coli*
ELISA: enzyme‐linked immunosorbent assay
ESAT‐6: early secreted antigenic target
ESX: ESAT‐6 secretory system
FTIR: Fourier‐transform infrared spectroscopy
HIV: human immunodeficiency virus
IDP: intrinsically disordered protein
IDR: intrinsically disordered region
IgG: immunoglobulin G
LPS: lipopolysaccharide
Mce: mycobacterial cell entry protein
MmpL: mycobacterial membrane protein large
PE: proline–glutamic acid
PGRS: polymorphic glycine‐rich sequence
PPE: proline–proline–glutamic acid
RD: region of difference
TLR2: Toll‐like receptor 2

*Mycobacterium tuberculosis* (*M.tb*), the etiological agent of tuberculosis (TB), causes millions of deaths worldwide. The emergence of drug resistance and co‐infection with HIV has further worsened the situation. The molecular mechanism(s) of pathogenesis and the associated host–pathogen interactions are yet to be elucidated, thereby hampering the development of new drugs or vaccines to target this bacterial pathogen. Thus, there is an immediate need for novel approaches to tackle this pathogen, which has coevolved with humans and thus possibly adapted well to counter host resistance 1.

*Mycobacterium tuberculosis *has undergone reductive evolution; that is, its genome size has decreased with gain in pathogenesis 2, 3. The only exception to this tendency is few protein families such as toxin anti‐toxin system and novel PE–PPE/PGRS family of proteins that are specific to mycobacteria. This gene family has expanded with the gain in pathogenesis and accounts for about 10% of the coding capacity of the *M.tb* genome 4, 5. Some of the genes are organized in a defined operonic pattern within the genome, where a PE gene is followed by a PPE gene 6 with few genes associated with members of the ESX family that are important virulence factors and T‐cell antigens. Many members of the PE–PPE/PGRS family are membrane attached and localize to the cell surface where they are involved in host–pathogen interactions. Some members of the family are involved in modulating the host immune response 7, 8, 9, 10, 11, 12, immune quorum sensing, and virulence 13, 14. Comparative genome analysis of *M.tb* H_37_Rv and BCG, a nonpathogenic attenuated strain of *Mycobacterium bovis,* revealed the absence of some genomic regions in BCG denoted as regions of difference (RDs). The exclusive presence of some RDs in pathogenic strains suggests that their encoded proteins are important virulence factors. There are 16 RDs, of which RD1 has been well studied. RD1 is absent in all the BCG strains, but present in all virulent strains where it codes for the *PE35, PPE68,* and *ESAT‐6* genes along with other genes belonging to the *ESX* secretory pathway. The relationship between RD1 and virulence of *M.tb* has been well established experimentally 15, 16. Therefore, exploring the structural flexibility and functional diversity of PE/PPE/PGRS proteins encoded within RDs could deliver novel insights into the molecular basis of pathogenesis employed by *M.tb*, particularly given the fact that only the PE25–PPE41 and PE8–PPE15 pairs have been crystallized from this family till now 17, 18.

The *M.tb* proteome has a relatively high content of intrinsically disordered proteins (IDPs) 19 primarily due to the PE–PPE/PGRS family proteins, which often contain long regions of structural disorder 20. Despite this, the role of protein disorder in *M.tb* pathogenicity and host–pathogen interactions is yet to be understood. Intrinsically disordered proteins (IDPs) and regions (IDRs) exist as ensembles of different conformations. Their conformational variability and adaptability, large interaction surface, numerous interaction motifs, and post‐translational modification sites allow them to participate in functions involving molecular recognition 21. Furthermore, the absence of structural constraints allows them to tolerate more mutations and hence contribute to faster rates of change during evolution. They are capable of moonlighting 22, promiscuous binding, and recognizing their targets with low affinity (transient binding) yet with high specificity 23, 24. Transition between the natively unfolded state and globular bound state provides a means for the thermodynamic regulation of IDP binding. Therefore, IDPs may confer advantages on *M.tb* such as hijacking the host pathways through molecular mimicry of peptide motifs 21 and promiscuous binding interactions 25, 26, as already determined in some viruses and bacterial pathogens 25, 27. They may also favor pathogen survival, both by inhibiting effective high‐affinity antibody response and possibly by interacting with host molecules necessary for attachment to and invasion of host cells.

Other *M.tb* protein families known to be associated with pathogenesis include the Mce (mammalian cell entry) and MmpL families (mycobacterial membrane protein large) 28. While the Mce proteins play an important role in the entry and survival of the bacteria in the host 29, the MmpL proteins are multisubstrate efflux pumps. We therefore selected the PE–PPE, PE–PGRS, Mce, and MmpL families, along with the entire secretome of *M.tb* 19 for analysis of structural disorder and potential interaction sites. Computational analysis revealed that PE–PGRS family had highest disorder along with some other PE and PPE family proteins. To complement our computational study, we unsuccessfully tried to clone, express, and purify PE–PGRS proteins. Considering the higher disorder content in some PE/PPE proteins, we further experimentally investigated two interacting protein pairs from the PE–PPE family, namely PE32/PPE65 and PE35/PPE68. Besides being disordered, these protein pairs are present within the RD regions and thus are suitable candidates to study the folding‐upon‐binding mechanism to gain insight into structure‐mediated pathogenicity. In our previous study, we demonstrated that the PE32–PPE65 protein complex is more immunogenic than its individual protein components 30. We now describe a step‐up approach to determine the role of structural flexibility in the functions of such PE–PPE protein pairs. Our results suggest that the presence of intrinsically disordered regions and host‐like interaction sites in *M.tb* proteins 31 could be a key strategy employed by the pathogen for rapid adaptive changes, host invasion, hijacking of host machinery, and diversifying the functions of its proteins.

## Materials and methods

### Dataset assembly

The *M.tb H37Rv* proteome was obtained from NCBI. The selected families were complemented by the PE10, PE11, PPE40, PE_PGRS63, and PE_PGRS58 sequences from TubercuList database (http://tuberculist.epfl.ch/), and the secretome was obtained from Ref. 19 (Table S1).

### Computational protein analysis

We predicted structural disorder for each member of the investigated families and reference set using the IUPred (long) algorithm 32. Residues with a predicted disorder propensity > 0.5 were considered as disordered. PE–PPE family disorder analysis was also carried out using the RONN prediction tool. For detecting disordered binding regions, we used the ANCHOR method 33.

### Expression and purification of proteins

The expression and purification of proteins were done according to previously published protocol 30, 34. Briefly, expression constructs of all four genes, PE32‐pET28a, PPE65‐pET28a, PE35‐pET28a, and PPE68‐pET28a, were transformed into *Escherichia coli* BL21(DE3) strain. Culture was induced with 1 mm IPTG for 3 h at 37 °C for the expression of recombinant proteins. Purification of recombinant proteins was carried out by sonicating bacterial cells and solubilizing recombinant proteins using 0.3% sarkosyl in phosphate‐buffered saline. Recombinant proteins were purified using Ni‐NTA affinity column and eluted with 200 mm imidazole. Eluted proteins were dialyzed for 24 h with buffer replaced at different time points with fresh (100 mm) phosphate‐buffered saline. Dialyzed proteins were concentrated using Centricons with 3 kDa cutoff. Protein was treated with polymyxin B agarose beads (Sigma, St. Louis, MO, USA) for the removal of endotoxins. Protein concentration was estimated using BCA protein estimation kit.

### Fluorescence spectra measurements

Fluorescence emission spectra of all four recombinant proteins (0.5 mg·mL^−1^) were carried out in a Jasco fluorometer (FP‐6200) (Jasco International Co., LTD, Tokyo, Japan). The excitation wavelength was set at 280 nm, and emission spectra were measured in the wavelength region 300–340 nm in quartz cuvette of path length 3 mm. All the experiments were carried out at 25 ± 0.1 °C with both excitation emission slits set at 5 nm. The fluorescence intensity of the blank solution was subtracted from that of each sample.

### FTIR spectroscopy

Protein samples of PE32, PE35, PPE65, and PPE68 were purified to high degree of purity (Fig. S1) and concentrated for successful FTIR experiments. To generate protein complex, both the proteins were mixed in equal amount in 1× PBS and incubated for 1 h at 4 °C. Proteins were stored at −20 °C for future use. Protein concentrations were estimated using BCA protein estimation kit. Phosphate‐buffered saline (100 mm) was used as protein storage and experimental buffer for FTIR. Concentrated 3 µL samples of the proteins were placed on a SensIR ATR reflection element. FTIR spectra were recorded at 4 cm^−1^ resolution on an Agilent Cary 630 spectrometer (Agilent Technologies, Inc., Santa Clara, CA, USA) equipped with a DTGS detector. The experiments were performed on the liquid sample at room temperature using the spectrometer software micro‐lab (Agilent Technologies, Inc., Santa Clara, CA, USA). For each sample, a 64‐scan single‐beam spectrum of the buffer was collected. For the protein sample, spectrum in the absorption mode (64 scans each) was recorded.

Secondary structure analysis was carried out by curve fitting using the opus software (Opus Consulting Solutions Inc. Alpharetta, GA, USA). The absorption bands at low wavenumber were free from features of noise as judged from the peaks above 1800 cm^−1^ and water vapor at below 1750 cm^−1^. A straight baseline passing through 1715 and 1600 cm^−1^ was subtracted before the curve fitting. The baseline was again modified by the least squares curve‐fitting program, which allows for a horizontal baseline to be adjusted as an additional parameter to obtain the best fit. The second‐derivative spectrum was used to determine the initial peak positions for curve fitting, and the peaks were fitted using Voigt functions 35, 36. In curve fitting, each component is a mixture of Lorentzian and Gaussian line shapes. The area under the entire band was considered 100%, and each component after fitting was expressed as a percent fraction.

### Immunization of mice

C57BL/6j mice (8–10 weeks age) were procured from National Institute of Nutrition, Hyderabad, India. The experimental protocol was approved by the Institute Animal Ethics Committee, and during all the procedures, Institute Animal Ethical Guidelines were followed. These animals were housed in plastic cages with wire lid and provided with access to unlimited pellet diets and water. A photoperiod of 12 h was provided. Experiment was performed in two parts: In one part, two groups of mice (five mice in each group) were immunized with individual proteins, and the third was taken as control; and in the second part, two groups were taken with same number of mice, one immunized with PE–PPE complex and the other taken as control. Mice were immunized subcutaneously with purified proteins (20 µg of protein in PBS) without any adjuvant in LPS‐free condition to minimize Th1/Th2 bias inherent to adjuvants. Purified proteins were passed through polymyxin B bead column to remove endotoxins. Mice injected with PBS served as controls. Injection was done subcutaneously at the base of the tail. A booster dose of respective proteins was given at the 15th day of primary immunization. Mice were sacrificed after 4 weeks of primary immunization.

### Serum collection, splenocyte isolation, and single‐cell preparation

Mice were euthanized, and blood was collected through cardiac puncture and transferred to a 1.5‐mL vial for coagulation at room temperature for 1 h. Later on, serum was collected by centrifugation of coagulated blood at 1500 ***g***. Spleens were isolated, crushed, and passed through a 40‐µm cell strainer to get a single‐cell suspension. Cells were pelleted down at 300 ***g*** for 10 min, washed with PBS, and resuspended in RBC lysis buffer. The process of resuspension of cells in RBC lysis buffer was repeated till there was no sign of RBC in the pellet. The cells were washed twice with PBS and once with incomplete DMEM and finally resuspended in DMEM containing 10% FBS.

### Splenocyte proliferation assay

Cells were seeded in 96‐well plates and treated with different concentrations of proteins (0.2, 1, and 2 µg·mL^−1^) for 3 days at 37 °C and 5% CO_2_. ^3^[H] thymidine (0.5 µCi·mL^−1^) was added to each well and incubated at 37 °C for 24 h. Cell harvester was utilized for harvesting the cells, and beta scintillation counter was used to measure cpm.

### Cell culture and ELISA for cytokine levels and B‐cell response

Splenocytes (0.1 × 10^6^ cells per well) were seeded in 96‐well plates in DMEM containing 10% FBS. Protein treatments with different concentrations were given for 72 h, and supernatants were collected and stored at −20 °C. Secreted cytokine levels in culture supernatant were measured using PeproTech (Rocky Hill, NJ, USA) standard ELISA development kits for TNF‐α, IL‐6, IL‐4, and IL‐10 and BioLegend ELISA MAX™ (San Diego, CA, USA) standard kit for IL‐1β. ELISA was carried out using the manufacturer’s protocol. Briefly, capture antibody was coated on 96‐well ELISA plates in PBS (PeproTech) or bicarbonate coating buffer and kept at room temperature or 4 °C overnight. Plates were washed with PBS‐T (0.05% Tween‐20) thrice. Three hundred microlitre of 1% BSA solution was used as blocking buffer, while 0.1% BSA was used as assay diluent. After 2 hours of blocking, the plate was washed four times with 300 µL of wash buffer and samples along with standards were added and incubated at room temperature for 2 h. Plates were again washed four times with wash buffer, and detection antibody was added. Plates were washed four times, and avidin‐conjugated antibody was added for 30 min at room temperature. After four washes, ABTS/TMB substrate was added for color development. 2 N H_2_SO_4_ was added to stop the reaction. For PeproTech kit, absorbance was measured at 405 nm, whereas for the BioLegend kit, absorbance was taken at 450 nm. B‐cell response against individual proteins was assayed using antibody against total IgG as described earlier 37.

### Intracellular cytokine staining and extracellular staining of surface markers

Splenocytes (1 × 10^6^) were seeded per well in 96‐well plates and restimulated with 10 µg·mL^−1^ proteins for 8 h in the presence of GolgiPlug™/GolgiStop^™^ (BD Biosciences, San Jose, CA, USA). Cells were harvested, washed, and stained for the CD4 and CD8 cell surface markers with anti‐CD4 and anti‐CD8 antibodies. Cells were fixed with 4% paraformaldehyde, permeabilized with 0.02% Triton X‐100, and then stained for intracellular IFN‐γ and IL‐2 (FITC for CD4, Alexa Fluor 488 for CD8, APC for IFN‐γ, and PE for IL‐2) Antibodies were procured from BD Biosciences.

### Statistical evaluation of immunological experimental results

prism 5 (GraphPad Software, San Diego, CA, USA) was used for statistical analysis, and the results were expressed as mean ± SD. One‐way ANOVA statistical comparisons were performed on the results to obtain *P* values. The value of *P* < 0.05 was considered statistically significant.

## Results

### PE–PPE/PGRS proteins of the three subfamilies are disordered to different extents

**Figure 1:**
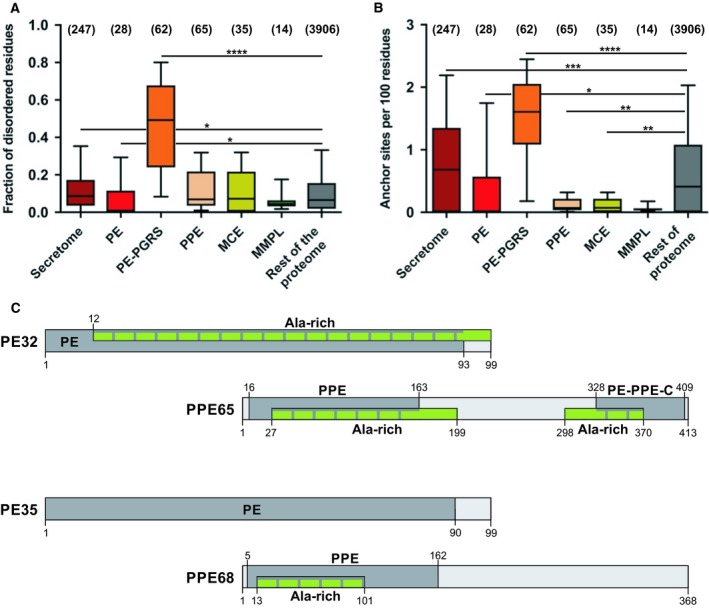
Computational analysis of *Mycobacterium tuberculosis* protein families and selected groups of proteins. We compared (A) the IUPred‐predicted protein disorder content and (B) density of ANCHOR binding sites of the selected six groups of proteins with the rest of the proteome. The number of proteins in each group is indicated above the respective box in brackets. Boxes contain 25–75% of the data with the median indicated as a horizontal line in the box. Lower and upper whiskers indicate 10% and 90% of the data, respectively. Significance was calculated by the Kruskal–Wallis test coupled with Dunn’s multiple comparisons post hoc tests for each investigated property, and significant differences are marked by stars (*P < 0.05; **P < 0.01, ***P < 0.001, ****P < 0.0001). (C) Domain maps of the shortlisted proteins for experimental study: PE32, PPE65, PE35, and PPE68 are depicted in light gray with Pfam entities shown as darker gray bars with the name and residue boundaries indicated. ExPASy‐PROSITE predicted ala‐rich low‐complexity regions marked in green.

Computational analysis by the IUPred and RONN disorder prediction methods revealed that the PE–PGRS subfamily is significantly more disordered than the other selected families or the rest of the proteome (Fig. 1A and Fig. S2). Composition bias is a common feature of intrinsically disordered proteins. These disordered proteins or disordered regions in proteins are usually enriched with charged amino acids such as Arg, Gln, Glu, Lys, Pro, and Ser and deficient in hydrophobic amino acids such as in Phe, Trp, Tyr, Cys, Ile, Leu, and Val. However, amino acids such as Ala, Gly, His, Asn, and Asp are known to be enriched in some intrinsically disordered proteins. Enrichment in disorder was also observed for secreted proteins that potentially modulate host cell processes through regulatory and signaling interactions. The same two groups of proteins were also enriched in ANCHOR‐predicted disordered binding sites (Fig. 1B), which are disordered patches likely mediating energetically favorable interactions with globular protein partners. While PE proteins were rather depleted in disordered regions, PPE proteins had similar disorder levels as the rest of the proteome. PE domain of PE proteins is around 100 residues in length, which attains an almost completely helical conformation in PE–PPE complexes [see Protein Data Bank (PDB) entry http://www.rcsb.org/pdb/search/structidSearch.do?structureId=4KXR]. As most of them probably show high helix propensities in their free forms, their low predicted disorder content is not surprising. PPE proteins are more variable in terms of their length and sequence. They all have an N‐terminal PPE domain that can form a stable complex with a specific PE partner domain. As a complex, they mediate interactions with components of the type VII secretion system 38. Besides their PPE domains, they have variable C‐terminal regions that can be short (as in PPE41), or might contain transmembrane regions (as in PPE37), other domains, or disordered regions (as in PPE37 and PPE68). Although the PPE family does not have a very high level of disorder on average, some of its members, like the ones investigated in this study, do have extended disordered regions that could play a major role in pathogenesis. In case of the MmpL proteins that are transmembrane efflux pumps and Mce membrane proteins, the low predicted disorder content and lack of ANCHOR binding sites are not surprising, as transmembrane regions generally receive very low disorder probability values.

### Computational analysis of the PE32, PPE65, PE35, and PPE68 proteins

Among the four proteins studied, PE32 and PPE65 are more structured according to IUPred (Fig. S3A). However, RONN predicted 15.1% and 54.96% structural disorder in these proteins, respectively (Fig. S3B). PE35 had little disorder, while PPE68 had a highly disordered C‐terminal region according to both IUPred and RONN, also interspersed with a number of ANCHOR binding sites (Fig. S3C,D). The interaction partners of the four proteins were checked in the IntAct 39 and STRING 40 databases. PE32 and PPE65 interact with each other, based on their genomic locations, and also with the EsxW and Esxl proteins. Besides interacting with each other and ESX‐1 specialized secretion system, proteins EsxA, B, H and Smn2, PE35, and PPE68 also bind the human TLR2 receptor 34. The proteins also have alanine‐rich low‐complexity regions and domains as shown in Fig. 1C and have a number of predicted high‐probability post‐translational modification sites, mostly N‐myristoylation and phosphorylation sites, the biological relevance of which is questionable. We also aligned the PE proteins, PE32 and PE35, to PE25 for which a structure is available in the PDB in complex with PPE41. The three proteins are identical in length but show remarkable sequence variation (Fig. S4A). The alignment of PPE65 and PPE68 to PPE41 shows that both PPE65 and PPE68 have long C‐terminal extensions compared to PPE41 that mainly consists of the PPE domain, and that these extensions do not show any detectable sequence similarity (Fig. S4B). The complete lack of sequence similarity outside the PPE domains of these proteins implies high rates of evolutionary changes and a high level of functional specialization within this subfamily.

### Protein–protein interaction studies using fluorescence spectroscopy

**Figure 2:**
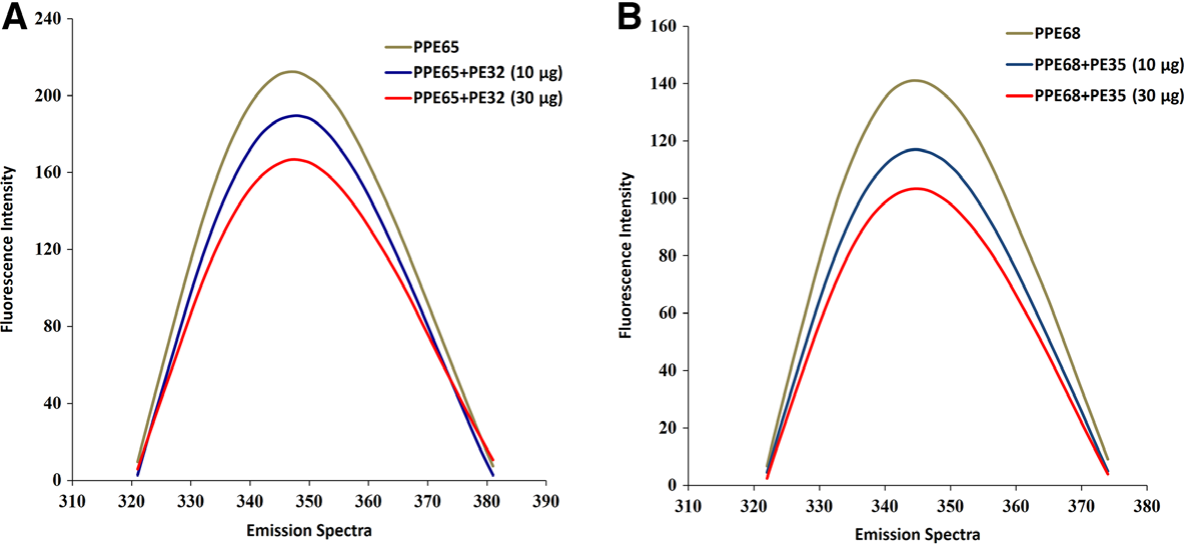
Fluorescence spectroscopy for protein–protein interactions: PE32–PPE65 and PE35–PPE68 pairs interact physically to form complex. (A) A clear shift in the emission spectrum of PPE65 (10 μg) was observed when different concentrations of PE32 (10 and 30 μg) were added. (B) A clear shift in the emission spectrum of PPE68 (10 μg) was observed when different concentrations of PE35 (10 and 30 μg) were added.

PE–PPE genes whose products form a functional protein complex are generally co‐operonic and thus co‐expressed 41. So far, PE25–PPE41 and PE8–PPE15 pairs have been copurified and cocrystallized 17, 18 probably due to the fact that PPE41 mainly consists of the PPE domain and lacks extended C‐terminal regions. We investigated the structures and interactions of two other pairs: PE32 and PPE65, as well as PE35 and PPE68. The proteins were expressed in *E. coli*, and recombinant proteins were purified as per earlier method 30. The purity of the proteins was checked through SDS/PAGE: Single bands were observed at their respective positions compared to the protein ladder (Fig. S1). As PE32 and PE35 proteins lack tryptophan residues, we used fluorescence spectroscopy to analyze the interaction of PE32 with PPE65 and PE35 with PPE68 (Fig. 2). A clear shift in the emission spectrum of PPE65 was observed when different concentrations of PE32 were added (Fig. 2A). The emission spectrum of PPE68 was similarly monitored while adding PE35 protein, and a pronounced change in fluorescence intensity was observed (Fig. 2B).

### Secondary structure analysis of PE32, PPE65, and the PE32–PPE65 complex

Infrared bands in amide I region (1700–1600 cm^−1^) arise mainly from the C=O stretching vibrations of the protein backbone. Different types of secondary structures give different C=O stretching vibrations in amide I region due to variations in molecular geometry and hydrogen bond patterns 42. For each protein sample, the spectrum was obtained against the buffer background. The protein absorbance spectrum was further checked with the buffer spectrum and subtracted in an iterative manner until a straight baseline was obtained in the 2000–1750 cm^−1^ spectral region. To resolve the extensively overlapping component bands in amide I region that arise from various secondary structural elements, second‐derivative analysis with 5‐point smoothing was applied. The data obtained from second‐derivative analysis were used to determine the number of bands (and their position) to resolve the protein spectrum into different secondary structure components. The band area for each component peak was used to calculate the relative contribution of that component to a particular protein’s secondary structure.

**Figure 3:**
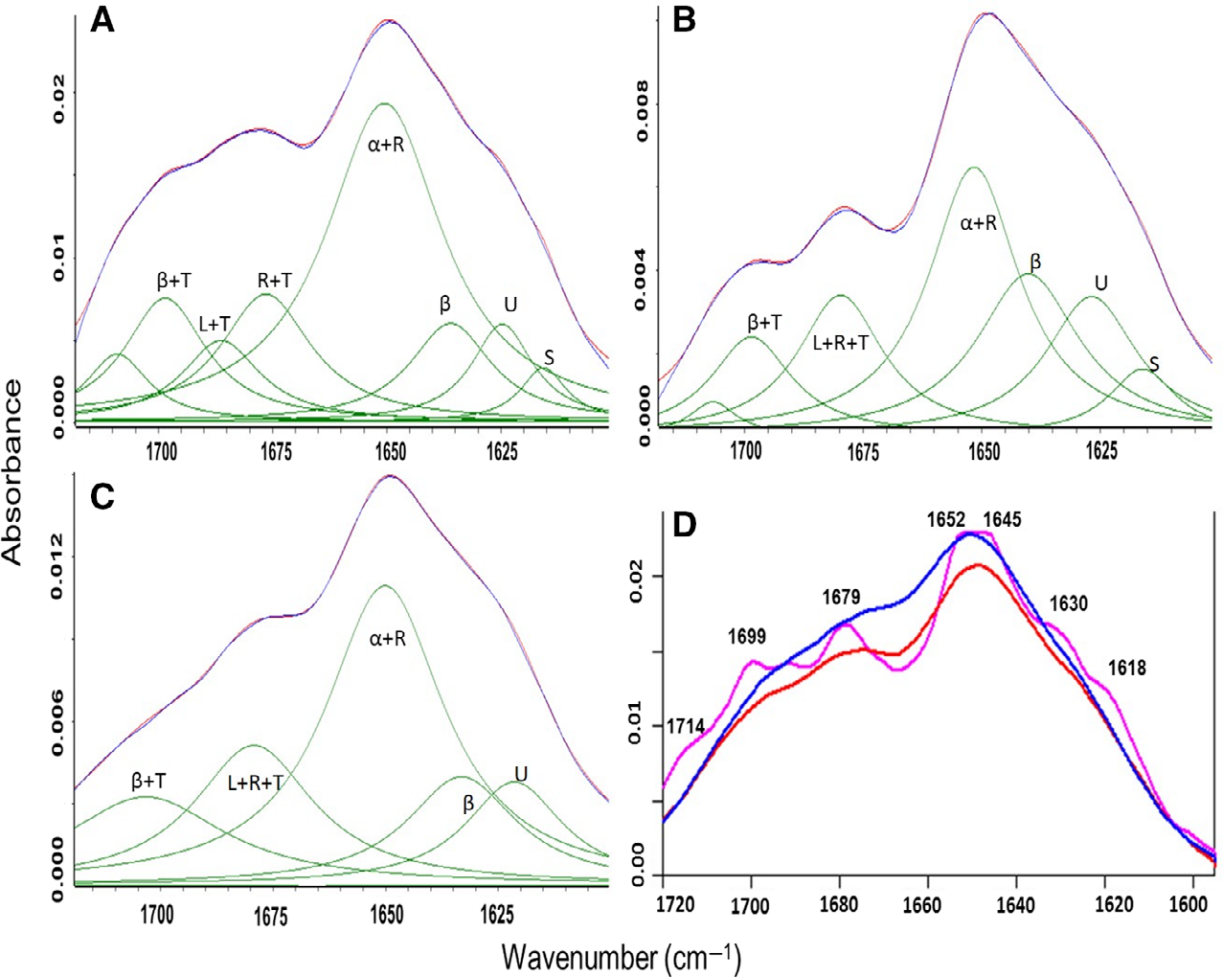
FTIR absorbance spectra of the PE32–PPE65 pair reveals structural shift: Curve fittings of the absorption spectrum of PE32, PPE65, and PE32–PPE65 are presented in A, B, and C, respectively. In the above curve fit, blue color depicts the original spectrum and red color indicates the quality of fit which results from protein secondary structural changes (green bands). Secondary structure content should be read as α: alpha helix; β: beta sheets; L: loops; T: turns; R: random coils; U: unstructured; and S: amino acid side chain. D represents average infrared absorption spectra of PE32, PPE65, and PE32–PPE65 complex and depicted in red, pink, and blue color, respectively.

Using curve‐fitting analysis on recombinant PPE65 (Fig. 3B), more than 66% of random coil and unstructured region content was observed, implying that the protein is loosely folded. The band observed below 1630 cm^−1^ (1630–1615 cm^−1^) indicated unstructured regions or protein aggregation. The bands near 1643 cm^−1^ (1650–1640 cm^−1^) and 1675 cm^−1^ (1680–1662 cm^−1^) arise from random coil conformations. The band near 1653 cm^−1^ (1660–1650 cm^−1^) signals alpha helical structure, while the band observed above 1680 cm^−1^ (1700–1680 cm^−1^) is assigned to beta sheets or beta turns. We also observed a fair amount of unstructured regions in PE32 (Fig. 3A); however, this protein was more structured in comparison with PPE65. When we mixed PE32 and PPE65 (in 1 : 1 ratio) to see the structural effects of the interaction, more than 66% ordered structure (36% alpha helices and 32% beta sheets) was observed. Thus, a structural shift from disorder to order occurred upon complex formation. However, significant amount of unstructured regions (> 30%) was still retained in the complex (Fig. 3C,D and Table S2), likely involving the C‐terminal half of PPE65. The band observed near 1709 cm^−1^ is tentatively assigned to Asp, Glu, or Asn, whereas the band at 1615 cm^−1^ is assigned to Asn. This indicates that these amino acid side chains play a key role in complex formation. These results also potentially explain why previous expression and purification attempts for most members of this family in isolation could be problematic, potentially precluding structural and biophysical studies.

### Secondary structure analysis of PE35, PPE68, and the PE35–PPE68 complex

**Figure 4:**
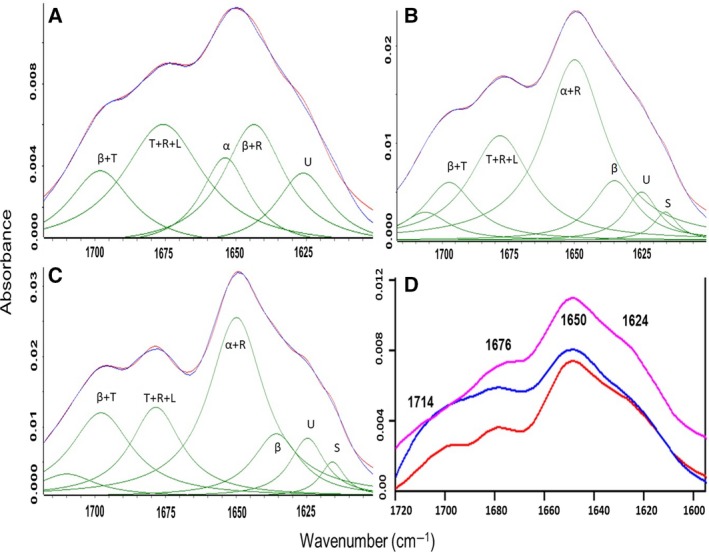
FTIR absorbance spectra of the PE35–PPE68 pair reveal an increase in structural order upon interaction: Curve fittings of the absorption spectrum of PE35, PPE68, and PE35–PPE68 are presented in A, B, and C, respectively. In the above curve fit, blue color depicts the original spectrum and red color indicates the quality of fit which results from protein secondary structural changes (green bands). Secondary structure content should be read as α: alpha helix; β: beta sheets; L: loops; T: turns; R: random coils; U: unstructured; and S: amino acid side chain. D represents average infrared absorption spectra of PE35, PPE68, and PE35–PPE68 complex and depicted in red, pink, and blue color, respectively.

The other co‐operonic protein pair, PE35 and PPE68, which were previously shown to interact with each other and the human TLR2 receptor, and to display increased immunogenic activity as a complex, was also used for FTIR analysis. PE35 displayed a highly disordered structure with 41% of regular secondary structures (Fig. 4A), to which alpha helices and beta sheets contributed 23% and 18%, respectively. The much longer PPE68 had a similar content of secondary structure (43%) as PE35, while in agreement with the applied disorder prediction methods, a significant amount of unstructured regions was also observed (Fig. 4B). Analysis of the structure content of the PE35–PPE68 complex revealed a major transition from disorder to order upon complex formation (Fig. 4C,D and Table S2), but with significant regions (40%) still retaining their disordered states in the complex, likely involving the C‐terminal disordered half of PPE68. The peak around 1714 cm^−1^ indicates the involvement of charged residues such as Asp/Glu/Asn/Gln in the interaction between the two proteins. In all, comparing the spectra of the individual proteins and the corresponding complexes showed major structural shifts toward a more structured state, but also indicated the presence of disordered regions in the complex state for both protein pairs.

### PE35 and PPE68 induce cell proliferation individually but suppress polyfunctional T cells

**Figure 5:**
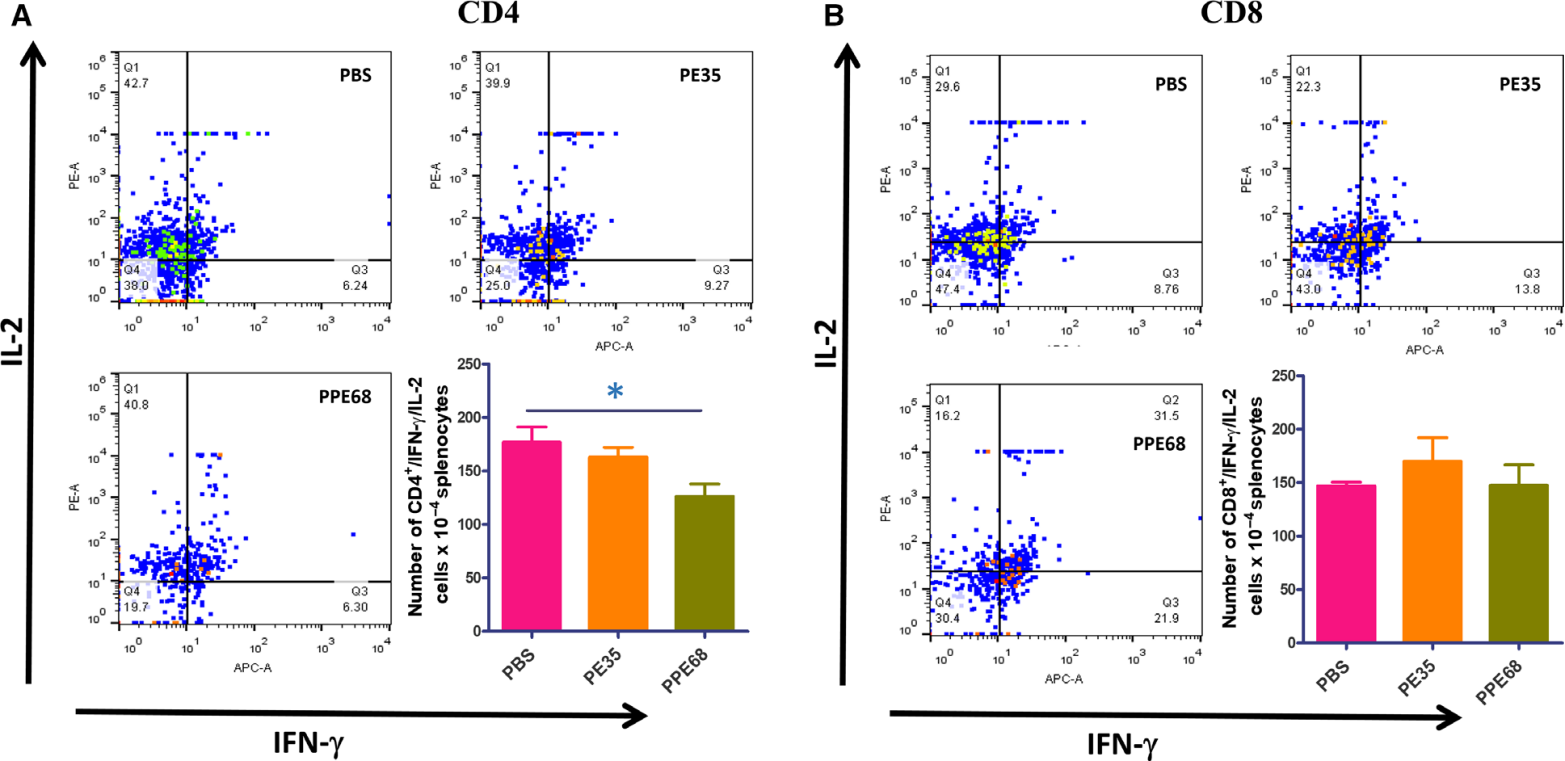
Individual PE35 and PPE68 proteins reduce the number of double‐positive CD4^+^ cells. Splenocytes were restimulated with 10 µg·mL^−1^ of recombinant proteins in culture for 8 h in the presence of GolgiPlug/GolgiStop solution (BD). Cells were washed and stained for intracellular IFN‐γ and IL‐2. (A) Individual PE35 and PPE68 proteins reduce the number CD4^+^ cells double‐positive for IFN‐γ and IL‐2. However, the numbers of CD8^+^ double‐positive for both were found to be unchanged. (B) Dot plots are the representative of experiments with four mice in each group. Statistical significance was determined by one‐way ANOVA, and data are presented as mean ± SD. *P* value < 0.05 was considered statistically significant.

To determine the functional significance of the biophysical features reported above, experiments were designed, based on animal immunization studies, to determine the immunogenicity of the individual proteins. Different concentrations of PE35 and PPE68 proteins (0.2, 1, and 2 µg·mL^−1^) were used to restimulate cultured splenocytes for 72 h which were then also treated with [H^3^] thymidine for 24 h. Harvested cells were used to count β activity using beta scintillation counter as a measure of cell proliferation. We found that proliferation in PPE68 restimulated cells was very high, while when applying a lower dose of PE35, it was not significant, implying that PE35 may be less antigenic. Clearly, individual PE35 and PPE68 proteins induced cell proliferation in splenocytes to different extents (Fig. S5). An earlier study with the PE35/PPE68 pair in macrophage cells has shown that these proteins suppress IL‐12 secretion but enhance IL‐10 secretion in a dose‐dependent manner 27. We restimulated splenocytes with the respective individual proteins in culture and determined the intracellular levels of cytokines (IL‐2 and IFN‐γ) through FACS. We observed a clear decrease in the number of IFN‐γ^+^/IL‐2^+^ CD4^+^ cells. However, the number of double‐positive CD8^+^ cells did not change significantly (Fig. 5A,B). These results from animal immunization experiments point to the low immunogenicity of individual proteins as opposed to the complex.

### The PE35–PPE68 complex displays enhanced downregulation of Th1 cytokines

**Figure 6:**
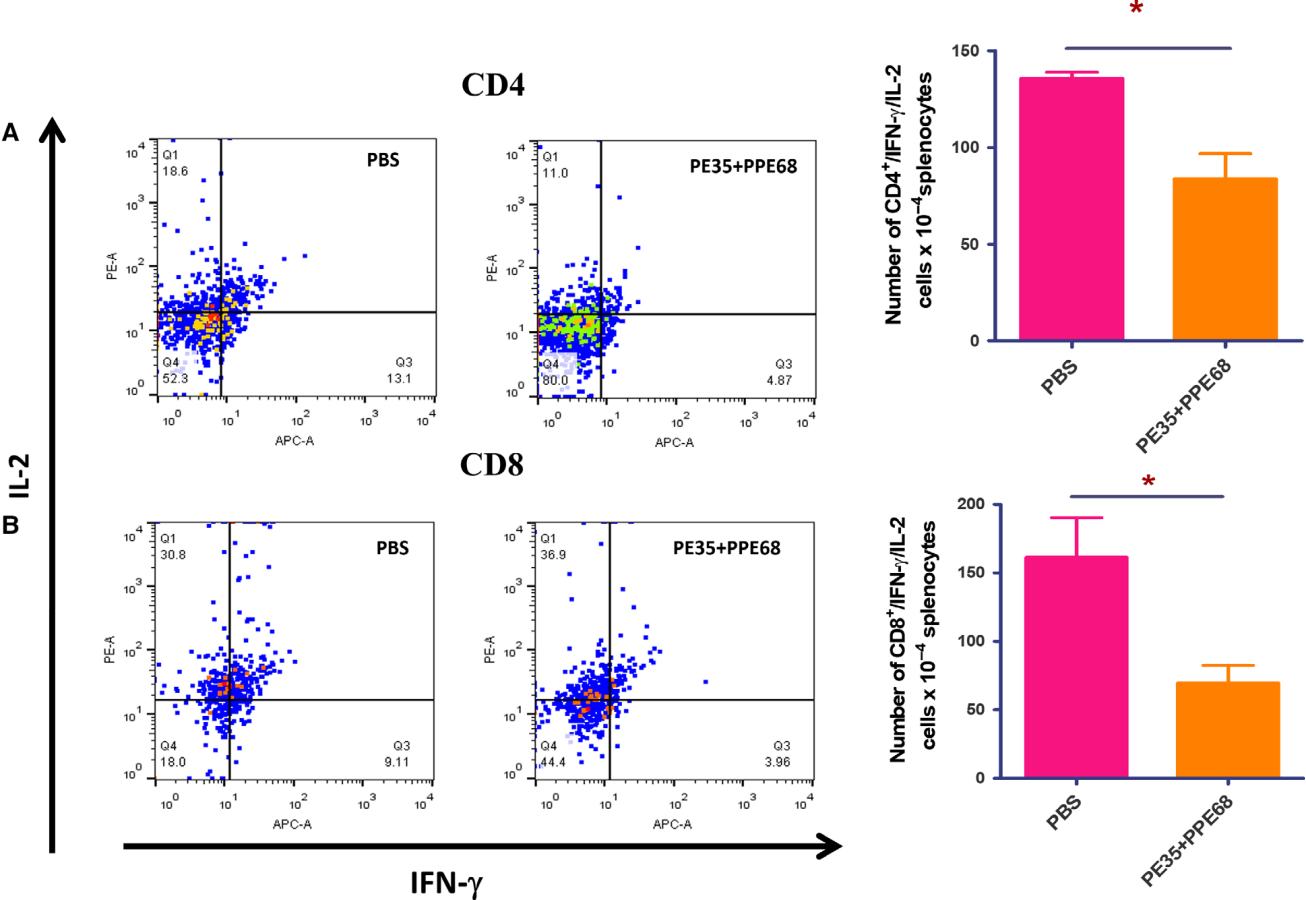
The PE35 and PPE68 protein complex markedly decreases the number of polyfunctional CD4^+^ and CD8^+^ cells. Splenocytes from PE35+PPE68‐immunized mice were restimulated with the complex of these two proteins in the presence of GolgiPlug/GolgiStop solution for 8 h. Cells were washed, harvested, and labeled for CD4/CD8 surface marker and intracellular IFN‐γ and IL‐2. (A1, A2) There is a decrease in the number of CD4^+^ cells double‐positive for IFN‐γ and IL‐2. (B1, B2) There is also a significant decrease in the number of CD8^+^ cells double‐positive for IFN‐γ and IL‐2. Dot plots are the representatives of experiments with four mice in each group. Statistical significance was determined by Student’s *t*‐test, and data are presented as mean ± SD. *P* value < 0.05 was considered statistically significant. *P < 0.05.

**Figure 7:**
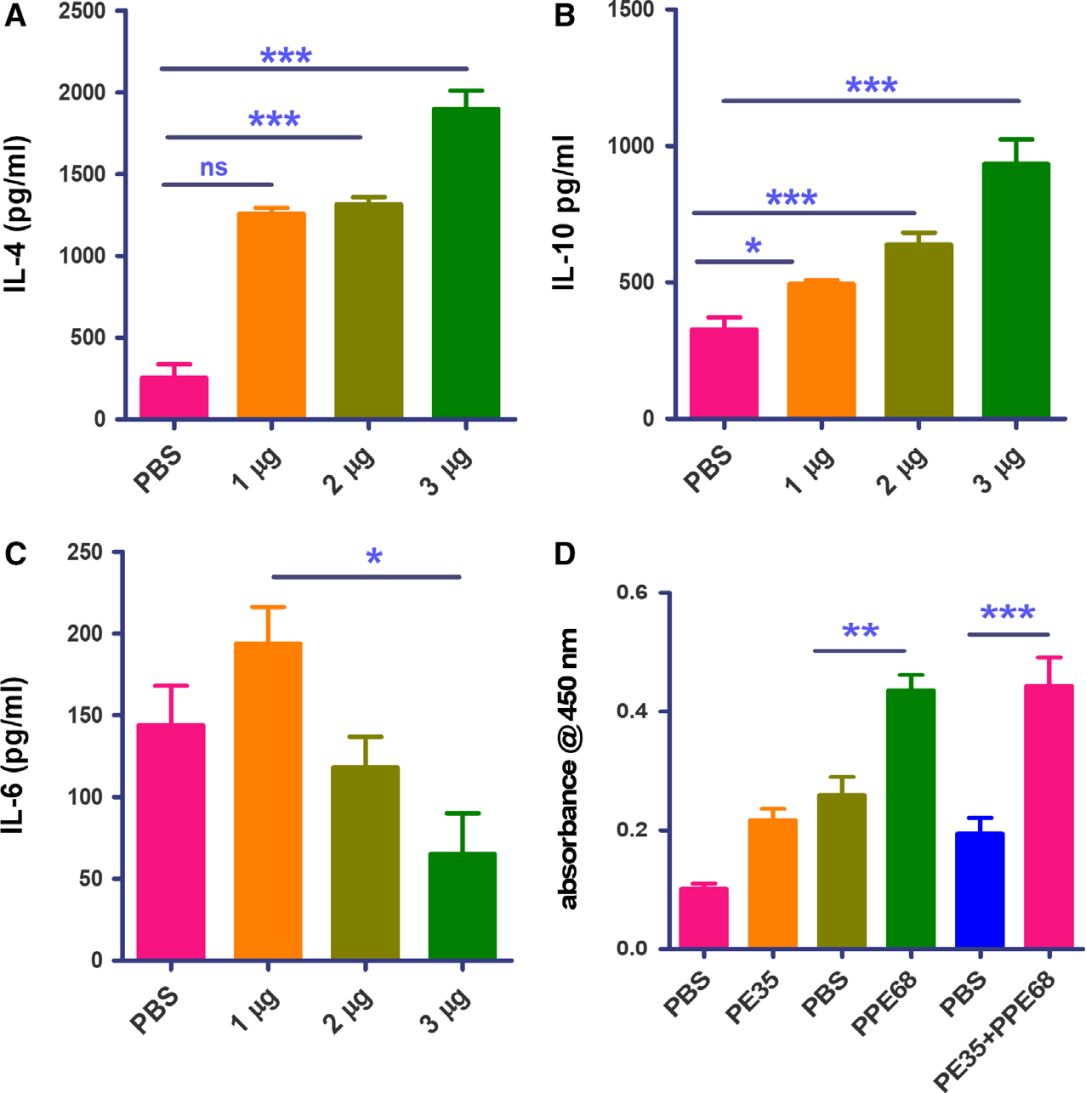
PE35 and PPE68 enhance secretion of Th2 cytokines in restimulated splenocytes. Splenocytes were restimulated with recombinant proteins in culture for 72 h. Supernatant from the culture was collected for sandwich ELISA. We observed a significant decrease in the level of secreted (A) IL‐4, (B) IL‐10, and (C) IL‐6 in a dose‐dependent manner. The change was significant between 1 and 3 µg·mL^−1^ of the proteins. Data represent experiments with four animals. Error bar represents SD, and one‐way ANOVA was used to calculate *P* value. A *P* value < 0.05 was considered significant. (D) B‐cell response against the PE35/PPE68 pair in mice. Serum collected from blood of immunized mice was used for performing ELISA for the level of total IgG against respective proteins. The level of IgG against PE35 was significant (lanes 1 and 2), while PPE68 was found to be a strong B‐cell antigen (lanes 3 and 4). The maximum level of IgG was found in the serum of mice immunized with both proteins (lanes 5 and 6). Data represent experiments with four animals. Statistical significance was determined by one‐way ANOVA, and data are presented as mean ± SD. *P* value < 0.05 was considered statistically significant. *P < 0.05, **P < 0.01, ***P < 0.001.

After investigating how the individual proteins modulate immune response, we investigated how the protein complex behaves. Interestingly, we observed a significant decrease in IFN‐γ^+^/IL‐2^+^‐double‐positive CD4^+^ and CD8^+^ cells when immunized with the complex of PE35–PPE68 (Fig. 6A,B). ELISA performed with the supernatant of restimulated splenocytes showed an increase in the level of IL‐4 and IL‐10 cytokines in a protein complex concentration‐dependent manner (Fig. 7A,B) with a significant decrease in the level of IL‐6 (Fig. 7C). These results demonstrate that the PE35–PPE68 complex shifts the balance toward Th2‐dominated immune response, favoring the survival of the pathogen inside the host.

### B‐cell response against PE35–PPE68 pair in mice

Earlier observations with a PE–PPE pair showed that the PE protein was less immunogenic 41. We predicted B‐cell antigenicity using the dnastar tool 43, which predicts PE35 to be less immunogenic, but PPE68 to be highly immunogenic with several antigenic patches (Fig. S6). Evaluation of the total IgG response against the respective proteins revealed no significant response against PE35 alone, significantly higher response against PPE68 alone, and an even higher response for the PE35–PPE68 complex (Fig. 7D).

## Discussion

Being an intracellular pathogen, *M.tb* potentially employs a repertoire of pathogenic effector proteins which can modulate host proteins to aid host cell entry and favor its survival inside macrophages. Given the fact that pathogenic *M.tb* strains have undergone reductive evolution 44, and based on our earlier observations 37, 41, we hypothesized that they might employ disordered proteins or disordered protein regions to ensure that their relatively small proteomes still have a sufficient level of functional diversity and potential to undergo adaptive changes for maintaining survival and pathogenesis. These disordered proteins utilize their inherent plasticity to adopt entirely different structure (and subsequently, function) upon binding to different partners, thus increasing the interactome without requiring multiple proteins. Pathogens are known to employ disordered proteins to disrupt and subvert the host cell cycle and normal cellular signaling processes 45. Emerging evidences have validated that the flexibility of proteins in other phyla also adds to their functionality particularly in context with multiple interactions. We have recently published the role of unstructured C terminus of mycobacterial PPE37 in host–pathogen interaction 31. A recent report also validates that secreted proteins, which are harbingers for interaction with host, from pathogens are also enriched in disordered amino acid stretches 46. Moreover, even in eukaryotic systems, the disorder in a protein is now considered a central characteristic for binding promiscuity to multiple ligands 47, 48.

Computational analyses revealed that the PE–PGRS subfamily is the most disordered among all the investigated pathogenesis‐related protein families and that the secreted proteins of the bacterium are also more disordered than the rest of its proteome. This relatively higher level of structural disorder in PE–PPE/PGRS proteins supports their anticipated functions in immune evasion and host–pathogen interactions, since many known pathogenic effector proteins are disordered. Disorder‐mediated aggregation propensity and susceptibility to proteolytic degradation make them very difficult to express and purify in recombinant form in isolation, leading to only the PE25–PPE41 and PE8–PPE15 pairs being successfully crystallized 17, 18. Thus, expansion of PE/PPE family in pathogenic mycobacteria seems justified as these proteins have accumulated multiple structural features that aid pathogenicity. The structural disorder is expected to enable these proteins as key signaling hubs that could modulate protein machinery of pathogen as well as host for its effective survival.

Biophysical studies revealed that PE35/PPE68 and PE32/PPE65 have unstructured regions to different extents and undergo disorder‐to‐order transition upon interaction. The observed infrared absorption bands from mainly charged amino acid side chains imply that they play a fundamental role in stabilizing the protein complex. The interaction of PE32 with PPE65 and PE35 with PPE68 also validates the biological relevance of the co‐operonic arrangement of their genes.

Our computational and experimental analyses suggest that while PE proteins show a considerable sequence variation, their short length and structural features seem relatively well‐conserved. We found that in their unbound forms, PE proteins are disordered to different extents, but based on the PE25–PPE41 complex structure and our observations, they appear to fold upon binding to their cognate PPE partner domains almost completely. On the other hand, PPE proteins are longer and only their N‐terminal PPE domains seem to take part in forming the complex with PEs and in interacting with Esx secretion system components 38. While the observed increase in secondary structure content upon interaction reaffirms that PE and PPE domains cofold and get stabilized in PE–PPE complexes, we have measured a relatively large unstructured content in both investigated complexes, implying that the C‐terminal variable halves of the PPE65 and PPE68 proteins retain their disordered states. This suggests the likelihood of multiple additional interactions that need to be understood to gain a holistic view of the mechanism of pathogenesis.

**Figure 8:**
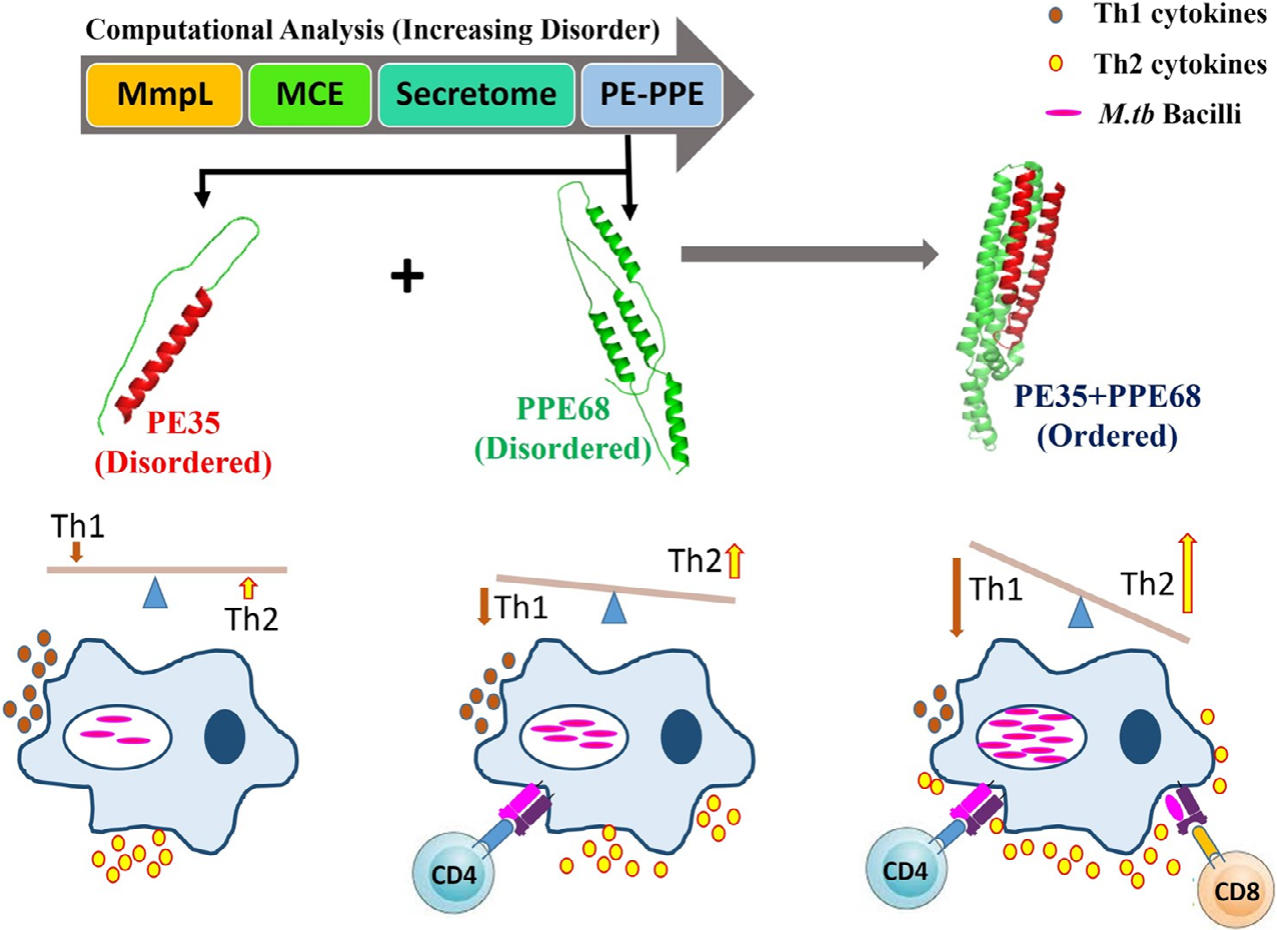
Proposed model describing the importance of IDRs in PE–PPE proteins. The PE35–PPE68 protein pair gains structural order on forming complex. By virtue of gain in structural order, complex shows enhanced immunomodulatory effect to enhance pro‐pathogen immune response. Even though PPE68 (green) protein individually downregulated Th1 response, the structurally ordered complex of PE35–PPE68 (Blue) has an enhanced effect on downregulation of Th1 protective cytokines. Th1 cytokines (brown) are decreased, while Th2 cytokines (yellow) are enhanced, which in turn could likely increase the survival chances of *M.tb.* This suggests that *M.tb* is able to counterbalance reductive evolution by employing this specific family of proteins, exploiting the disorder‐to‐order transition. Being exposed on the pathogen surface and showing high levels of functional specialization among family members through variable segments, PE–PPE protein complexes successfully influence the host immune system toward favoring pathogen survival.

This transition toward structural order upon interaction of PE and PPE suggests gain of function. Investigating the immunogenicity of the proteins demonstrated that PE35 and PPE68 are antigenic, with PPE68 emerging as a strong B‐cell antigen. PE35 proved to be a weak B‐cell antigen, in agreement with earlier observations regarding the PE counterparts of other PE–PPE partners and with the PE domain of PE–PGRS33 49. PE/PPE proteins help in the formation of the complex required for transport of the proteins across the membrane 18, 50. Our results, however, showed that PE35/PPE68 plays an important role in immune modulation, which was earlier also reported for PE32/PPE65 30. The complex of PE35–PPE68 displayed gain of function by exhibiting significantly higher antibody response in mice, along with significantly enhanced suppression of IFN‐γ^+^/IL‐2^+^‐double‐positive CD4^+^ and CD8^+^ cells. A significant increase in the level of IL‐4 and IL‐10 cytokines along with a significant decrease in the level of IL‐6 in the supernatant of restimulated splenocytes was evident in a protein dose‐dependent manner when using the complex of PE/PPE for immunization as well as for *in vitro* stimulation. These observations indicate that PE–PPE complexes modulate immune response toward Th2 type, leading to increased anti‐inflammatory and reduced pro‐inflammatory cytokine production that eventually aids the escape/survival of the bacterium within the host. Although individual proteins were also immunogenic and displayed anti‐inflammatory behavior, the stable PE–PPE complex had a greater impact. Therefore, the observed structural transition expectedly led to the observed gain of function that could be one of the contributing factors for enhanced pathogenicity (Fig. 8). Earlier observations on PE/PPE also depicted additive immunological effect as a pair 34, 37, and we now provide a biophysical perspective of the observed effect. The stability accorded to the complex enables modulation in interaction with host proteins to enhance virulence. This could have profound implications in vaccine‐based and other pathogenesis‐related studies as disorder‐to‐order content in a protein is likely to affect the possible outcome of the interaction.

In summary, infection and intracellular survival strategies of *M.tb* may partly rely on disordered proteins, as its pathogenesis‐related PE–PPE/PGRS proteins are enriched in structurally disordered regions. The repetitive and disordered nature of PE–PPE/PGRS proteins likely enables fast evolutionary changes and thus may mask the pathogen from host immune response, similar to what has been described for merozoite surface protein 2 (MSP2) of the malaria pathogen *Plasmodium falciparum* 51. Our results suggest that *M.tb* is able to counterbalance reductive evolution by employing this specific family of proteins, many of which contain disordered regions. These disordered regions achieve a more ordered state upon PE–PPE interaction or likely retain partially disordered states even after the formation of the specific complex. Being exposed on the pathogen surface and showing high levels of functional specialization among family members through variable segments, PE–PPE protein complexes successfully influence the host immune system toward favoring pathogen survival. Furthermore, the presence of multiple pairs/variants of such complexes 38 in the pathogenic toolbox of *M.tb* likely allows for using them in alternative combinations on its cell surface, thereby further hampering effective host immune response. Future studies should aim at a better understanding of the role of disordered proteins in *M.tb,* which will, in turn, help in deciphering the molecular mechanisms of the most prevalent infectious disease, and in developing improved strategies for its effective control.

## Conflict of interest

The authors declare no conflict of interest.

## Author contributions

JA, JAS, MK, and RP performed the experiments. NZE, MMB, and SEH designed the project. JA, MK, MMB, NZE, RP, and SEH helped in writing the manuscript. JAS, SK, AS, NZE, and SEH helped in the interpretation of results.

## Supporting information


**Fig. S1.** SDS/PAGE image of the purified PE32, PPE65, PE35, and PPE68 proteins.
**Fig. S2.** Structural analysis of the PE–PPE/PGRS protein family using RONN. Each subfamily was divided into three groups, i.e., completely disordered members with more than 80% disorder, partially disordered members with disorder between 20–80% and ordered proteins having less than 20% structural disorder.
**Fig. S3.** Structural disorder predictions for PE32, PE35, PPE65, and PPE68. (A) Predicted IUP red disordered regions and disordered binding sites using the ANCHOR tool in PE32 and PPE65. (B) Predicted disordered regions in PE32 and PPE65 using RONN. (C) Predicted IUP red disordered regions and disordered binding sites in PE35 and PPE58 using ANCHOR. (D) Disordered regions in PE35 and PPE68 as predicted by the RONN tool.
**Fig. S4.** Alignments of our investigated PE and PPE proteins to those with available complex structure. The (A) PPE41, PPE65 and PPE68 proteins as well as the (B) PE25, PE32 and PE35 proteins were aligned using Clustal Omega 1.2.4. The regions of the PPE and PE domains are marked by light grey and yellow backgrounds, respectively.
**Fig. S5.** Individual recombinant PE35 and PPE68 proteins induce cell proliferation. Different concentrations of individual recombinant proteins were used for re‐stimulating the splenocytes in culture for7 2 hrs. ^3^[H] thymidine (0.5 mci/ml) was added to each well and incubated at 37 °C for 24 hours. Cells were harvested and β scintillation counter was employed for counting Beta activity. Beta activity was significant for the cells re‐stimulated with 1 μg/ml or higher dose of PE35 while it was significant for 0.2 μg/ml or higher dose of PPE68, suggesting PPE68 to be more antigenic. Data represents experiments with three mice in each group. Statistical significance was determined by one‐way ANOVA and data represented as mean ± SD. *p *value <0.05 was considered statistically significant.
**Fig. S6.** Antigenicity index of PE35 and PPE68. Antigenicity plots depicting the antigenic regions of PE35 and PPE68.
**Table S1.** Identifiers of the protein family members and secretome proteins used in the analyses.
**Table S2.** Secondary structure content in the investigated recombinant proteins and their complexes in percentage.
