# Supplementary material for: Disorder‐to‐order transition in PE–PPE proteins of Mycobacterium tuberculosis augments the pro‐pathogen immune response
Source: FEBS Open Bio. 2019 Dec 17;10(1):70–85. doi: 10.1002/2211-5463.12749 (PMC6943233; doi:10.1002/2211-5463.12749)
Supplement: Supplementary file 1 — Fig. S1. SDS/PAGE image of the purified PE32, PPE65, PE35, and PPE68 proteins. Fig. S2. Structural analysis of the PE–PPE/PGRS protein family using RONN. Each subfamily was divided into three groups, i.e., completely disordered members with more than 80% disorder, partially disordered members with disorder between 20–80% and ordered proteins having less than 20% structural disorder. Fig. S3. Structural disorder predictions for PE32, PE35, PPE65, and PPE68. (A) Predicted IUP red disordered regions and disordered binding sites using the ANCHOR tool in PE32 and PPE65. (B) Predicted disordered regions in PE32 and PPE65 using RONN. (C) Predicted IUP red disordered regions and disordered binding sites in PE35 and PPE58 using ANCHOR. (D) Disordered regions in PE35 and PPE68 as predicted by the RONN tool. Fig. S4. Alignments of our investigated PE and PPE proteins to those with available complex structure. The (A) PPE41, PPE65 and PPE68 proteins as well as the (B) PE25, PE32 and PE35 proteins were aligned using Clustal Omega 1.2.4. The regions of the PPE and PE domains are marked by light grey and yellow backgrounds, respectively. Fig. S5. Individual recombinant PE35 and PPE68 proteins induce cell proliferation. Different concentrations of individual recombinant proteins were used for re‐stimulating the splenocytes in culture for7 2 hrs. 3[H] thymidine (0.5 mci/ml) was added to each well and incubated at 37 °C for 24 hours. Cells were harvested and β scintillation counter was employed for counting Beta activity. Beta activity was significant for the cells re‐stimulated with 1 μg/ml or higher dose of PE35 while it was significant for 0.2 μg/ml or higher dose of PPE68, suggesting PPE68 to be more antigenic. Data represents experiments with three mice in each group. Statistical significance was determined by one‐way ANOVA and data represented as mean ± SD. p value <0.05 was considered statistically significant. Fig. S6. Antigenicity index of PE35 and PPE68. Antigenicity plots [file FEB4-10-70-s001.pdf]

Figure S1

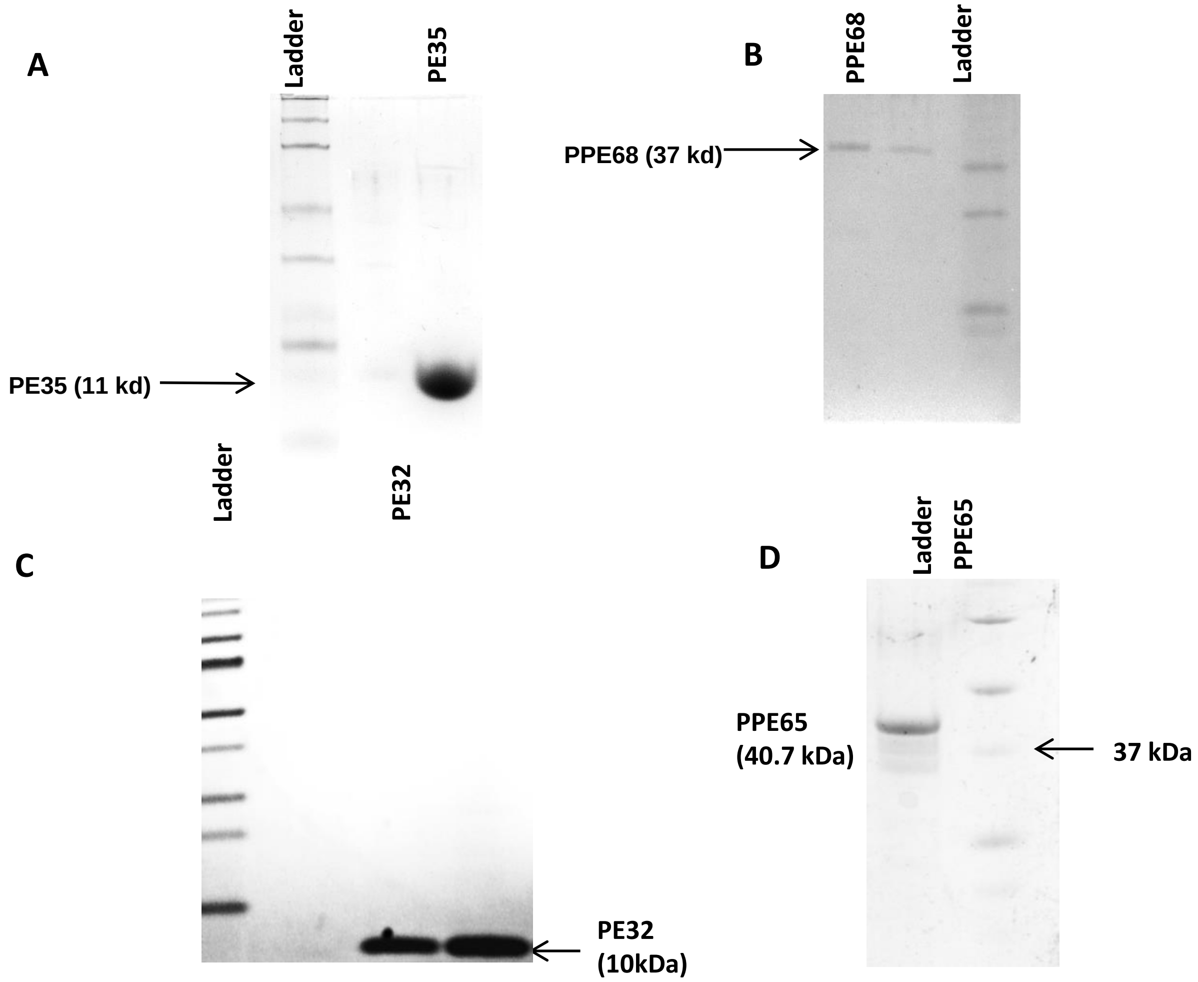

Figure S1: SDS-PAGE image of the purified PE32, PPE65, PE35 and PPE68 proteins.

Figure S2

	Disordered (80-100%)	Partially Disordered (20-80%)	Ordered (0-20%)
PPE(69)		65	4
PE(35)	1	32	2
PE_PGRS(64)	31	31	3

Figure S2: Structural analysis of the PE-PPE/PGRS protein family using RONN.
Each subfamily was divided into three groups, i.e. completely disordered members with more than 80% disorder, partially disordered members with disorder between 20-80% and ordered proteins having less than 20% structural disorder

Figure S3

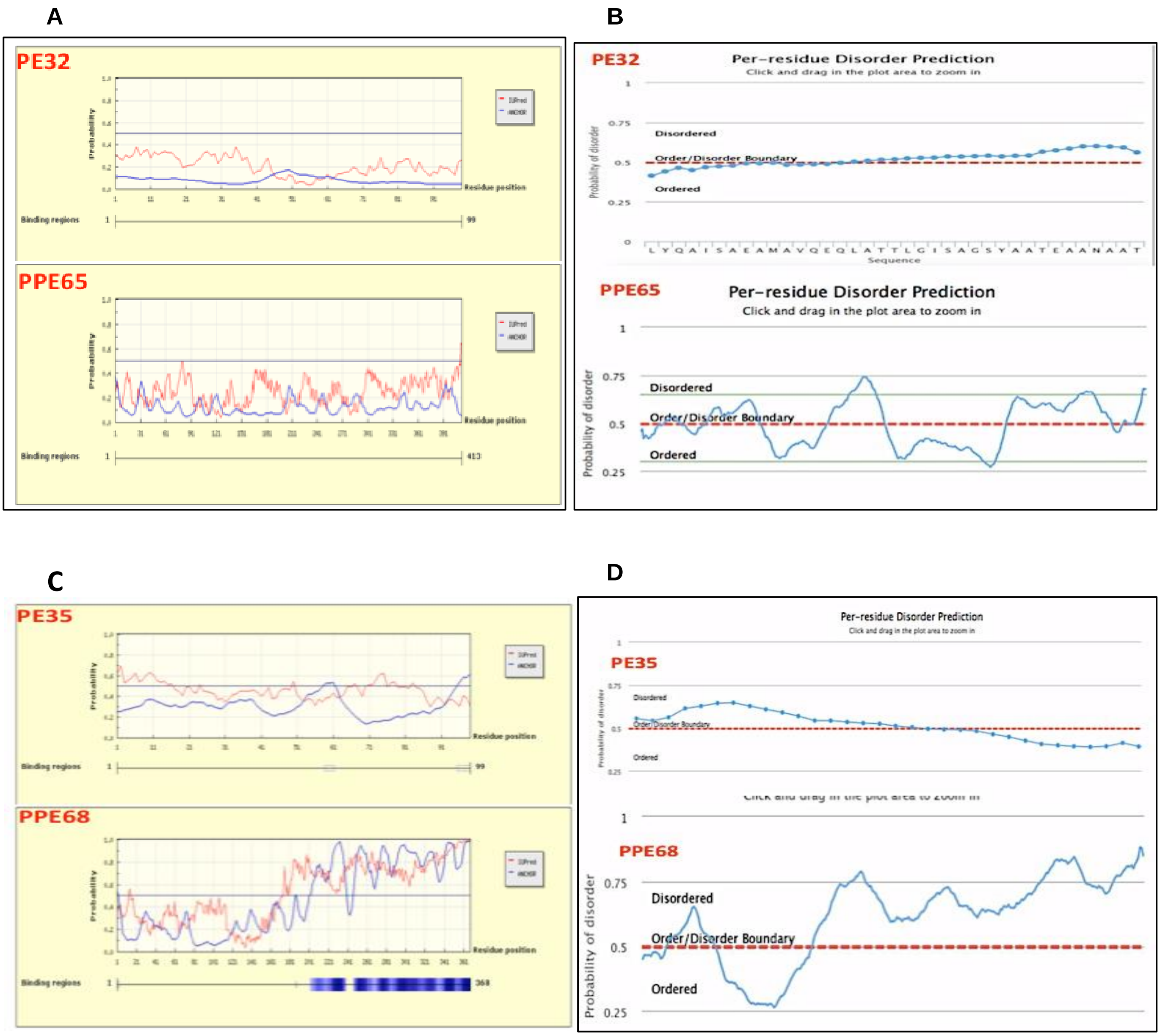

Figure S3: Structural disorder predictions for PE32, PE35, PPE65 and PPE68: (A) Predicted IUPred disordered regions and disordered binding sites using the ANCHOR tool in PE32 and PPE65. (B) Predicted disordered regions in PE32 and PPE65 using RONN. (C) Predicted IUPred disordered regions and disordered binding sites in PE35 and PPE58 using ANCHOR. (D) Disordered regions in PE35 and PPE68 as predicted by the RONN tool.

Figure S4

(A)

```
CLUSTAL O(1.2.4) multiple sequence alignment
PE25_MYCTU MSFVITNPEALTVAATEVRRIRDRAIQSDAQVAPMTTAVRPPAADLVSEKAATFLVEYAR 60
PE32_MYCTU MSIMHAEPEMLAATAGELQSINAVARAGNAAVAGPTTGVVPAAADLVSLLTASQFAAHAQ 60
PE35_MYCTU MEKMSHDPIAADIGT-QVSDNALHGVTAGSTALTSVTGLVPAGADEVSAQAATAFTSEGI 59
*. : :* : :: . ..: . .*.: * .** ** :*: :. .
PE25_MYCTU KYRQTIAAAAVVLEEFAHALTTGADKYATAEADNIKTFS- 99
PE32_MYCTU LYQAISAEAMAVQEQLATTLGISAGSYAATEAANAATIA- 99
PE35_MYCTU QLLASNASAQDQLHRAGEAVQDVARTYSQIDDGAAGVFAE 99
* * .. . :: * .*: : .::
```

(B)

```
CLUSTAL O(1.2.4) multiple sequence alignment
PPE41_MYCTU -MHFEAYPPEVNSANIYAGPGPDSMLAAARAWRSLDVEMTAVQRSFNRTLLSLM-DAWAG 58
PPE65_MYCTU MLDFAQLPPEVNSALMYAGPGSGPMLAAAAAWEALAAELQTTASTYDALITGLADGPWQG 60
PPE68_MYCTU -MLWHAMPPELNTARLMAGAGPAPMLAAAAGWQTLSAALDAQAVELTARLNSLG-EAWTG 58
: : ***:*:* : ** * ***** .*.:* . : : : .* * *
PPE41_MYCTU PVVMQLMEAAKPFVRWLTDLCVQLSEVERQIHEIVRAYEWAHHDMVPLAQIYNNRAERQI 118
PPE65_MYCTU SSAASMVAAATPQVAWLRSTAGQAEQAGSQAVAAASAYEAAFFATVPPPEIAANRALLMA 120
PPE68_MYCTU GGSDKALAAATPMVVWLQTASTQAKTRAMQATAQAAAYTQAMATTPSLPEIAANHITQAV 118
. : **.* * ** . * . * . ** * :* *:
PPE41_MYCTU LIDNNALGQFTAQIADLDQEY-DDFWDEDGEVMRDYRLRVSDALSKLTPWKAPPPIAHST 177
PPE65_MYCTU LLATNFLGQNTAAIAATEAQY-AEMWAQDAAAMYGYAGASA-AATQLSPFNPAAQTINPA 178
PPE68_MYCTU LTATNFFGINTIPIALTEMDYFIRMWNQAALAMEVYQAETAV-NTLFEKLEPMASILDPG 177
* .* :* * ** : :* :* : . .* * : : : : .
PPE41_MYCTU VLVAP---VSP---ST--ASSRTDT----------------------------------- 194
PPE65_MYCTU GLASQAASVGQAVSGAANAQALTDIPKALFGLSGIFT-----NEPPWLT-DLGKALGLTG 232
PPE68_MYCTU ASQS--------------------TTNPIFGMPSPGSSTPVGQLPPAATQTLGQLGEMSG 217
:
PPE41_MYCTU ------------------------------------------------------------
PPE65_MYCTU HTWSSDGSGLIVGGVLGDFVQGVTGSAELDA-SVAMDTFGKWVSPARLMVTQFKDYFGLA 291
PPE68_MYCTU PMQQLTQPLQQVTSLF-SQVGGTGGGNPADEEAAQMGLLGTSP----LS----------N 262
PPE41_MYCTU ------------------------------------------------------------
PPE65_MYCTU HDLPKWASEGAKAAGEAAKALPAAVPAIPSAGLSGVAGAVGQAASVGGLKVPAVWTATTP 351
PPE68_MYCTU H--PLAGGSGPSA-G---------AGLLRAESLPGAGGSLTRTPLMSQLI----EKPVAP 306
PPE41_MYCTU ------------------------------------------------------------
PPE65_MYCTU AASPAVLAASNGLGAAAAAEGSTHAFGGMPLMGSGA-GRAFNNFAAPRYGFKPTVIAQPP 410
PPE68_MYCTU SVMPAAAAGSS-------------ATGGAAPVGAGAMGQGAQSGG----STRPGLVAPAP 349
PPE41_MYCTU -------------------
PPE65_MYCTU AGG---------------- 413
PPE68_MYCTU LAQEREEDDEDDWDEEDDW 368
```

Figure S4: Alignments of our investigated PE and PPE proteins to those with available complex structure. The (A) PPE41, PPE65 and PPE68 proteins as well as the (B) PE25, PE32 and PE35 proteins were aligned using Clustal Omega 1.2.4. The regions of the PPE and PE domains are marked by light grey and yellow backgrounds, respectively.

Figure S5

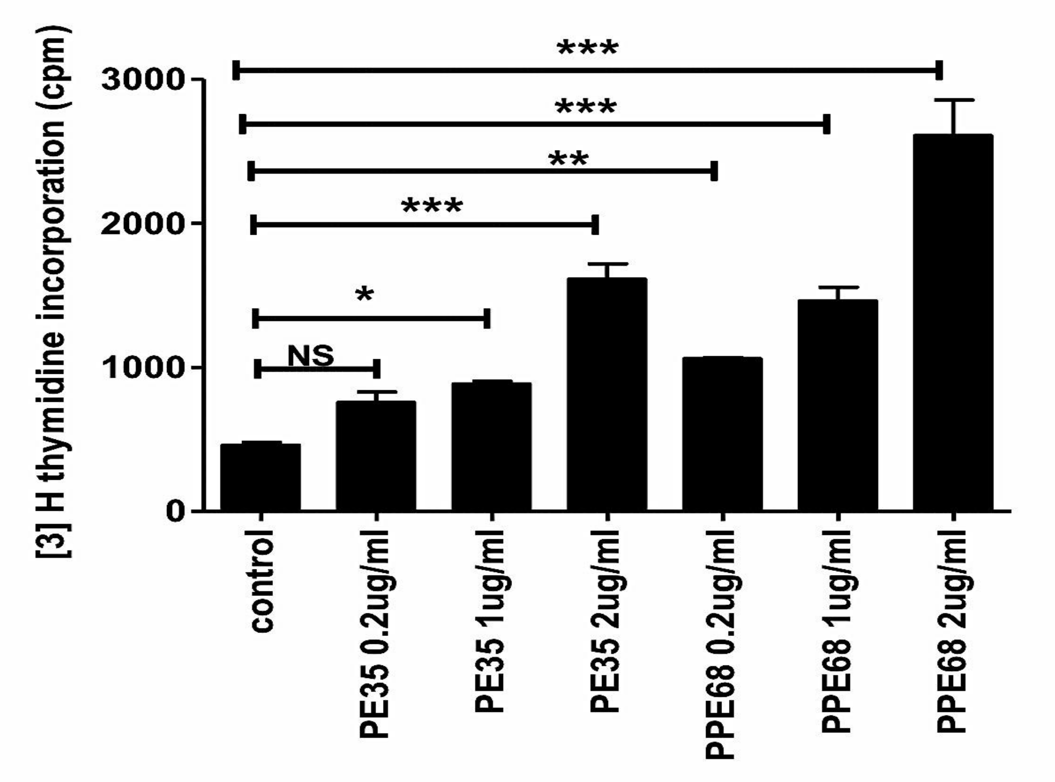

Figure S5: Individual recombinant PE35 and PPE68 proteins induce cell proliferation. Different concentrations of individual recombinant proteins were used for re-stimulating the splenocytes in culture for 72 hrs. 3[H] thymidine (0.5mci/ml) was added to each well and incubated at 37o C for 24 hours. Cells were harvested and β scintillation counter was employed for counting Beta activity. Beta activity was significant for the cells re-stimulated with 1 µg/ml or higher dose of PE35 while it was significant for 0.2 µg/ml or higher dose of PPE68, suggesting PPE68 to be more antigenic. Data represents experiments with three mice in each group . Statistical significance was determined by one-way ANOVA and data represented as mean ± SD . *p* value <0.05 was considered statistically significant.

Figure S6

PE35

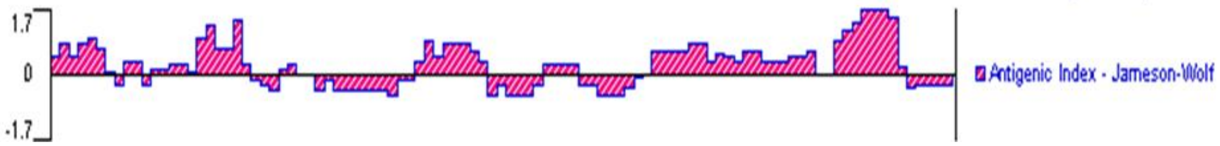

PPE68

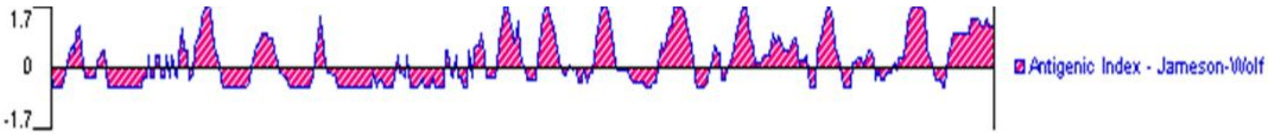

Figure S6: Antigenic index of PE35 and PPE68. Antigenicity plots depicting the antigenic regions of PE35 and PPE68.

Supplementary Table S1: Identifiers of the protein family members and secretome proteins used in the analyses.

PE subfamily

Gene Id	Protein name	Gene Id	Protein name	Gene Id	Protein name
YP_177690.1	PE1	YP_177794.1	PE13	YP_177882.1	PE25
YP_177696.1	PE2	YP_177797.1	PE14	YP_177907.1	PE27
YP_177697.1	PE3	YP_177805.1	PE15	YP_177685.1	PE29
YP_177710.1	PE5	YP_177810.1	PE16	YP_177975.1	PE31
YP_177717.1	PE6	YP_177825.1	PE17	YP_177999.1	PE32
YP_177766.1	PE7	YP_177834.1	PE18	YP_178000.1	PE33
YP_177779.1	PE8	YP_177837.1	PE19		
YP_177784.1	PE9	YP_177843.1	PE20		
CCP43841.1	PE10	YP_177858.1	PE22		
YP_177792.1	PE11/LIPX	YP_177867.1	PE23		
YP_177793.1	PE12	YP_177880.2	PE24		

PPE subfamily

Gene Id	Protein name	Gene Id	Protein name	Gene Id	Protein name
YP_177690.1	PPE1	YP_177830.1	PPE24	YP_177918.1	PPE46
YP_177704.1	PPE2	YP_177833.1	PPE25	YP_177932.1	PPE49
YP_177709.1	PPE3	YP_177835.1	PPE26	YP_177934.1	PPE50
YP_177711.1	PPE4	YP_177836.1	PPE27	YP_177935.1	PPE51
YP_177714.1	PPE5	YP_177839.1	PPE28	YP_177936.2	PPE52
YP_177715.1	PPE6	YP_177840_1	PPE29	YP_177937.1	PPE53
YP_177720.1	PPE7	YP_177841.1	PPE30	YP_177960.1	PPE54
YP_177721.2	PPE8	CCP44573.1	PPE31	YP_177963.1	PPE55
YP_177726.2	PPE10	YP_177844.1	PPE32	YP_177964.1	PPE56
YP_177727.1	PPE11	YP_177845.1	PPE33	YP_177971.1	PPE57
YP_177753.1	PPE12	YP_177655.1	PPE34	YP_177972.1	PPE58
YP_177764.1	PPE13	YP_177850.1	PPE35	YP_177973.1	PPE59
YP_177765.1	PPE14	YP_177859.1	PPE36	YP_177976.1	PPE60
YP_177778.1	PPE15	YP_177861.1	PPE37	YP_177984.1	PPE61
YP_177790.1	PPE16	YP_177870.1	PPE38	YP_177985.1	PPE62
YP_177791.1	PPE17	YP_177871.1	PPE39	YP_177987.1	PPE63
YP_177795.1	PPE18	CCP45144.1	PPE40	YP_177988.1	PPE64
YP_177801.1	PPE19	YP_177881.1	PPE41	YP_177998.1	PPE65
YP_177806.1	PPE20	YP_177893.1	PPE42	YP_178009.1	PPE66
YP_177817.1	PPE21	YP_177906.1	PPE43	YP_178010.1	PPE67
YP_177827.1	PPE22	YP_177677.1	PPE44	YP_178022.1	PPE68
YP_177828.1	PPE23	YP_177913.1	PPE45	YP_178024.1	PPE69

PE_PGRS subfamily

Gene Id	Protein Name	Gene Id	Protein Name	Gene Id	Protein Name
YP_177692.1	PE_PGRS1	YP_177799.1	PE_PGRS24	YP_177909.1	PE_PGRS48
YP_177693.1	PE_PGRS2	YP_177809.1	PE_PGRS25	YP_177961.2	PE_PGRS49
YP_177707.1	Rv0278c	YP_177811.1	PE_PGRS26	YP_177962.1	PE_PGRS50

YP_177708.1	PE_PGRS4	YP_177812.1	PE_PGRS27	YP_177965.1	PE_PGRS51
YP_177713.1	PE_PGRS5	YP_177813.1	PE_PGRS28	YP_177968.1	PE_PGRS52
YP_177736.1	PE_PGRS6	YP_177814.1	PE_PGRS29	YP_177978.1	PE_PGRS53
YP_177739.1	PE_PGRS7	YP_177826.1	PE_PGRS30	YP_177979.1	PE_PGRS54
YP_177749.1	PE_PGRS8	YP_177832.1	PE_PGRS31	YP_177980.1	PE_PGRS55
YP_177750.1	PE_PGRS9	YP_177842.1	PE_PGRS32	YP_177981.2	PE_PEGRS56
YP_177751.1	PE_PGRS10	YP_177846.1	PE_PGRS33	YP_177982.1	PE_PGRS57
YP_177752.1	PE_PGRS11	YP_177847.1	PE_PGRS34	YP_177993.1	PE_PGRS58
YP_177759.1	PEPGRS12	YP_177854.1	PE_PGRS35	YP_177994.1	PE_PGRS59
YP_177760.1	PE_PGRS13	YP_177862.1	PE_PGRS37	YP_178001.1	PE_PGRS60
YP_177761.1	PE_PGRS14	YP_177865.1	PE_PGRS38	YP_178002_1	PE_PGRS61
YP_177763.1	PE_PGRS15	YP_177869.1	PE_PGRS39	YP_178019_1	PE_PGRS62
YP_177773.1	PE_PGRS16	YP_177875.1	PE_PGRS40	YP_177924.1	PE_PGRS63
YP_177774.1	PE_PGRS17	YP_177878.1	PE_PGRS41		
YP_177775.1	PE_PGRS18	YP_177886.1	PE_PGRS42		
YP_177780.1	PE_PGRS19	YP_177887.1	PE_PGRS43		
YP_177781.1	PE_PGRS20	YP_177891.1	PE_PGRS44		
YP_177783.1	PE_PGRS21	YP_177895.1	PE_PGRS45		
YP_177786.1	PE_PGRS22	YP_177896.1	PE_PGRS46		
YP_177798.1	PE_PGRS23	YP_177902.1	PE_PGRS47		

Mce family

YP_177700.2		YP_177740.1	RV0589	NP_214686.1	Rv0172
NP_215100.1		YP_177852.1	RV1966	NP_215106.1	Rv0592
NP_216479.1	Rv1963c	NP_218015.1	RV3498C	NP_216486.1	Rv1970
NP_218012.1	Rv3495c	YP_177701.1	RV0169	NP_214687.1	Rv0173
NP_214680.1	Rv0166	NP_216483.1	Rv1967	NP_215107.1	Rv0593
NP_215101.1	Rv0587	YP_177977.1	Rv3499c	NP_216487.1	Rv1971
NP_216480.1	RV1964	NP_214684.1	Rv0170	NP_214688.1	Rv0174
NP_218013.1	Rv3496c	NP_216484.1	Rv1968	NP_215108.1	Rv0594
NP_214681.1	Rv0167	NP_218017.1	Rv3500c	NP_218011.1	Rv3494c
NP_215102.1	Rv0588	NP_214685.1	Rv0171		
NP_216481.1	Rv1965	NP_215105.1	Rv0591		
NP_218014.1	Rv3497c	NP_216485.1	Rv1969		
NP_214682.1	Rv0168	NP_218018.1	Rv3501c		

MmpL family

NP_214716.1	Rv0202c	NP_216073.1	Rv1557)	NP_215699.1	Rv1183
NP_215021.1	Rv0570	NP_218340.1	Rv3823c	NP_215662.1	Rv1146
NP_216038.1	RV1522C	NP_214916.1	Rv0402c		
NP_217458.1	RV2942C	NP_215661.1	Rv1145		
NP_214720.1	Rv0206c	NP_216855.1	Rv2339		
NP_215190.1	Rv0676c	NP_214964.1	Rv0450c		

Secretome

Gene Id	Gene id	Gene id	Gene id	Gene id
NP_214684.1	NP_215391.1	NP_217510.1	NP_215954.1	NP_215868.1
NP_214686.1	YP_177768.1	NP_217520.1	NP_215964.1	YP_177647.1
NP_214688.1	YP_177769.1	NP_217522.1	NP_215965.1	NP_216245.1
NP_214864.1	YP_177770.1	NP_217532.1	NP_215970.1	NP_216245.1
NP_215077.1	NP_215511.1	NP_217552.1	NP_215991.1	NP_216320.1
NP_215993.1	NP_215553.1	NP_217560.1	NP_216328.1	NP_216326.1
NP_216424.1	NP_215554.1	NP_217709.1	NP_216349.1	NP_216331.1
NP_216448.1	NP_215591.1	NP_217710.1	NP_216353.1	NP_216343.1
YP_177855.1	NP_215682.1	YP_177950.1	NP_216385.1	NP_216407.1
NP_216706.1	NP_215690.1	NP_217761.1	NP_216392.1	NP_216422.1
NP_216815.1	NP_215699.1	NP_217919.1	NP_216584.1	NP_216590.1
NP_217935.1	NP_215713.1	NP_218012.1	NP_216626.1	NP_216656.1
NP_218276.1	NP_215714.1	NP_218101.1	NP_216708.1	NP_216830.1
NP_218363.1	NP_215768.1	NP_218104.1	NP_216716.1	NP_216885.1
YP_177694.1	NP_215785.1	NP_218146.1	NP_216717.1	NP_217147.2
NP_214756.1	NP_215786.1	NP_218146.1	NP_216736.1	NP_217339.1
NP_214758.1	NP_215887.1	NP_218210.1	NP_216752.1	NP_217547.1
NP_214950.1	NP_215898.1	NP_218277.1	NP_216757.3	NP_217784.1
NP_215590.1	NP_215951.1	NP_218352.1	NP_216767.1	NP_217871.1
NP_215839.1	NP_216004.1	NP_218391.1	YP_177868.1	NP_217886.1
NP_216402.1	NP_216057.1	YP_178023.1	NP_216961.1	NP_218001.1
NP_217347.1	YP_177838.1	NP_218435.2	YP_177884.1	NP_218144.1
YP_178017.1	NP_216361.1	NP_217945.1	NP_217188.1	NP_218222.1
NP_218321.1	YP_177849.1	YP_177710.1	YP_177905.1	NP_218239.2
NP_214516.1	NP_216400.1	YP_177727.1	NP_217364.1	NP_218398.1
NP_214523.1	NP_216415.1	YP_177805.1	NP_217390.1	NP_218416.1
NP_214568.1	NP_216426.1	YP_177831.1	NP_217622.1	NP_215301.1
NP_215145.1	NP_216427.1	YP_177881.1	YP_177927.1	NP_215514.1
NP_217717.1	NP_216442.1	YP_177882.1	NP_217674.1	NP_215935.1
NP_214526.1	NP_216496.1	YP_178021.1	NP_217765.1	NP_216403.1
NP_214554.2	NP_216500.1	YP_177689.1	NP_217827.1	NP_216917.2
NP_214578.1	NP_216576.1	NP_214577.1	NP_217873.1	NP_217549.1
NP_214630.1	NP_216596.1	NP_214580.1	NP_217914.1	NP_217930.1
NP_214687.1	NP_216596.1	NP_214589.1	NP_218002.1	NP_218008.1
NP_214717.1	NP_216769.1	NP_214639.1	NP_218026.1	NP_218089.1
NP_214733.1	NP_216817.2	NP_214725.1	NP_218185.1	
YP_177705.1	NP_216862.1	NP_214805.1	NP_218185.1	
NP_214797.1	NP_216892.1	NP_214829.1	NP_218227.3	
NP_214801.1	NP_216966.1	NP_214877.1	NP_218242.1	
NP_214823.1	NP_217060.1	NP_214922.1	NP_218358.1	
NP_214912.1	NP_217079.1	NP_214976.1	NP_218431.1	
NP_214916.1	NP_217091.1	NP_215050.2	NP_214529.1	
NP_214925.1	NP_217092.1	NP_215040.1	NP_214986.1	

NP_214991.1	NP_217101.1	NP_215358.1	NP_215004.1	
NP_214965.1	NP_217184.1	NP_215366.1	NP_215497.1	
NP_215073.1	NP_217209.1	NP_215399.1	NP_214533.1	
NP_215097.1	NP_217237.1	NP_215566.1	NP_214654.1	
NP_215191.1	NP_217315.1	YP_177782.1	YP_177617.1	
NP_215194.1	NP_217389.1	NP_215614.1	NP_214706.1	
NP_215246.1	NP_217391.1	NP_215614.1	NP_214854.1	
NP_215288.2	NP_217391.1	NP_215677.1	NP_214969.1	
NP_215350.1	NP_217421.1	NP_215931.1	NP_215188.1	
NP_215353.1	YP_177914.1	NP_215952.1	NP_215674.1	
NP_215382.1	NP_217461.1	NP_215953.1	NP_215702.1	

Supplementary Table S2: Secondary structure content in the investigated recombinant proteins and their complexes in percentage

Recombinant protein	1615-1625 (unstructured /AAS)	1630-1640 (beta sheets)	1640-1650 & 1660-1680 (random coil/loop)	1650-1660 (alpha helices)	1680-1705 (turn/beta sheets)
PE32	16	10	25	32	17
PPE65	18		48	28	16
PE32&PPE65 complex	16	12	16	36	20
PE35	19	15	25	23	18
PPE68	24		33	29	14
PE35&PPE68 complex	10	18	20	32	20
